# Myosin Class I Genes Follow a Cell Type-Specific Transcription Pattern in Human Haematopoietic Cell Lines

**DOI:** 10.3390/ijms27041777

**Published:** 2026-02-12

**Authors:** Alexandra E. Golysheva, Anna V. Tvorogova, Artyom A. Tyukaev, Aleena A. Saidova, Daria M. Potashnikova

**Affiliations:** 1 Department of Cell Biology and Histology, School of Biology, M.V. Lomonosov Moscow State University, 119234 Moscow, Russia; alexandra_golysheva@mail.ru (A.E.G.);; 2 School of Bioengineering and Bioinformatics, M.V. Lomonosov Moscow State University, 119234 Moscow, Russia; artyomtyukaev@gmail.com; 3 Biology Department, Shenzhen MSU-BIT University, Shenzhen 517182, China

**Keywords:** myosin class I, human myosins I, transcriptome analysis, qPCR

## Abstract

Class I myosins form an abundant group of single-headed motor proteins that interact with actin and membrane phospholipids and are employed in a variety of cell processes like cell shape remodeling, motility and invasion, exocytosis, and endocytosis. The large number of class members and their uneven distribution in human cells and tissues complicate their study. Furthermore, this hinders the assessment of their biomarker potential and mechanistic role in oncogenesis and other diseases. Despite the emerging data on their mutations and altered abundance in a number of tumors and other diseases, most studies examine class I myosins individually or in groups of several proteins. Thus, the question of their redundancy and possible functional substitution is ignored. In this work, we simultaneously assess all eight human *MYO1* genes on cell lines of different origins by RNA-seq re-analysis. We also report a qPCR system for human myosin I screening and test it on a set of 35 continuous cell lines widely used in biomedicine and fundamental research. These data clarify the restricted expression patterns of *MYO1* genes in various human cell types and allow to assess the pattern specific of haematopoietic cell lines.

## 1. Introduction

Myosin class I motor proteins first described in 1973 in *Acanthamoeba castellanii* [1] belong to an abundant group of non-muscle, structurally similar, single-headed myosins that perform a range of functions within cells. To date, eight class I myosins encoded by *MYO1A*, *MYO1B*, *MYO1C*, *MYO1D*, *MYO1E*, *MYO1F*, *MYO1G*, and *MYO1H* genes have been found in higher vertebrates, including humans [2,3]. Additionally, a number of splice variants have been described for some of them: the data on gene localization and the available sequences for humans are summarized in Supplementary Table S1. The general structure shared by all of myosin I proteins includes three major domains: the head, the neck, and the tail. The head domain is highly conserved and possesses ATPase and actin binding activity [3]. The kinetics of the motor head ATPase varies between class I myosins [4], thus implying functional differences between them. The neck domain contains one or several regulatory calmodulin-binding IQ motifs [5]. Myosins I differ in the number of IQ motifs: myosins IE and IF have one IQ motif; myosins IH, ID, and IG have two IQ motifs; while myosins IC, IA, and IB may have three and more IQ motifs [5,6,7,8]. The tail of every myosin I contains an obligatory tail homology (TH1) domain that incorporates a pleckstrin homology (PH) domain responsible for interactions with anionic phospholipids like phosphoinositides of the plasma membrane and the intracellular vesicles [9,10]. The two optional tail domains (TH2 and TH3 (SH3)) are found in the so-called long-tailed myosins IE and IF and ensure the interactions with membrane proteins or cytoskeleton [11,12]. The range of binding partners for myosins I implies a substantial input of this whole class into membrane remodeling processes, maintaining membrane tension, cell motility, and endo- and exocytosis. Whether the structural differences between individual myosin I proteins change the repertoire of their binding partners and how the binding preferences change their respective functions within cells is still to be thoroughly studied.

The available data suggest that mammalian class I myosins are distributed in a tissue-specific manner. For instance, myosin 1A was detected as the most abundant in the enterocytes, specifically in the brush border [13], where it maintains the proper morphology of the microvilli linking the plasma membrane to the actin core of the microvillus and directing the membrane movement along the actin bundles [14,15]. Myosin IB appears in virtually all tissues [8,16], especially in the liver, lungs, heart, and brain. Similarly, myosin IC is expressed in most tissues; however, different isoforms of this protein demonstrate different distribution patterns [16,17]. Its cytoplasmic isoform is tissue-specific [17], while the nuclear myosin IC isoform is ubiquitously expressed [18] and presumably participates in chromatin modifications that influence gene transcription and cell cycle progression [19]. Myosin ID is expressed in heart, spleen, kidney, lung, liver, and, most notably, in the central and peripheral nervous system [20]. It is found along the axons and is involved in myelination and remyelination [21,22]. The expression of myosin ID in tracheal epithelial cells is associated with maintaining the basal bodies’ alignment and the unidirectional movement of the cilia [23]. Myosin IE is expressed in podocytes and plays an important role in maintaining normal tissue morphology, supporting cell-to-cell adhesions and normal glomerular filtration [24]. Apart from that, it supports immune cell activation, spreading, motility, and cytokine secretion, as was shown for B-lymphocytes and macrophages [25,26]. Myosins IF and IG both are predominantly expressed in hematopoietic cells [27,28] and selectively detected in the spleen, mesenteric lymph nodes, and thymus. Myosin IF induces the formation of filopodia in macrophages/monocytes, maintains their adhesions and their polarization to M1 pro-inflammatory phenotype [29,30], and also influences the adhesion and migration of intraepithelial lymphocytes [31]. Myosin IG is expressed by both T- and B-lymphocytes [32] and localizes in their membrane protrusions, and also in macrophages where it drives FcγR-mediated phagocytosis [33]. Little is known about myosin IH; however, its elevated expression was demonstrated in the testes and locally in the neural tissue [34,35]. Even though some of these findings have to be verified for humans, the emerging view is that a considerably large number of different myosin I class members are unevenly distributed within the organism. Unfortunately, many of the findings so far focus on individual class I myosins or several of them and do not provide the full view of these proteins’ representation, let alone their isoforms. This complicates further research into their role, cooperation, and interchangeability in the cell.

The apparent redundancy of myosin class I proteins that is often being overlooked becomes very evident in mouse knock-out studies. For instance, the *Myo1a* knock-out leads to brush border defects, decreased levels of lipid raft proteins and myosin IE, but increased levels of myosin IC [14]. Another study showed that myosin ID is also expressed in the terminal part of the microvillus and changes localization upon the *Myo1a* knock-out [36]. This cumulatively may explain the lack of overt phenotype in the whole animal. This example shows that a complex assessment of class I myosins’ expression is required to fully understand the adaptive changes in the cell and the changes that cannot be compensated for and thus lead to disease.

The role of class I myosins in human pathological processes is rapidly being revealed with major focus on oncogenesis. The prognostic value of myosin IA in human colon cancer was established along with its role as tumor suppressor in this model. The decreased expression rate of this protein is associated with unfavorable prognosis and the lower disease-free and overall survival of the patients [37]. Conversely, myosin IB is upregulated in colon cancer compared to the adjacent normal mucosa and healthy epithelium. It is associated with tumor progression in patients and with enhanced cell motility, metastatic potential, and cytoskeleton rearrangement in human cell models [38,39]. Apart from colorectal carcinoma, myosin IB expression is elevated in cervical cancer [40] and also in the head and neck squamous cell carcinoma (HNSCC), where it is associated with metastasis and poor prognosis [41,42]. Myosin IC is elevated in liver fibrosis and in nonalcoholic fatty liver disease (NAFLD) in humans and is associated with further hepatocellular carcinoma (HCC) in mice [43]. It was demonstrated that this myosin sensitizes liver tissue to induced fibrotization. Myosin ID gene methylation helps distinguish malignant and benign skin spitzoid tumors [44]; however, no transcriptional studies have yet been implemented. Overexpression of myosin IE was detected at transcriptomic level and associated with bad prognosis in lung and breast carcinoma [45,46]. Translocations involving the *MYO1F* gene are associated with acute monocytic leukemia [47]. The impact of this mutation in leukemogenesis is yet unknown. Hyperexpression of *MYO1G* has been identified in pediatric patients with acute lymphoblastic leukemia and associated with bad prognosis and relapse after remission [48,49]. This implies the role of myosin IG in leukemia pathogenesis. No association with tumors have yet been revealed for myosin IH, although its mutations have been identified as biomarkers for spastic spinal paraplegia and congenital central hypoventilation syndrome [35,50]. Thus, a growing body of evidence suggests a role of class I myosins in human diseases; however, the whole pattern of their expression is still lacking in most cases. A comprehensive approach to simultaneous class I myosins’ detection would bring a new vision of their redundancy, sufficiency, co-operation, and unique functions and thus improve our understanding of their role in health and disease. Here, we evaluate the expression of all class I myosin genes in an array of model cell lines in silico, through RNA-seq, and in vitro, through qPCR, to define cell type-specific patterns for human haematopoietic cell lines.

## 2. Results

### 2.1. In Silico Analysis of Myosin Class I Proteins

**Figure 1:**
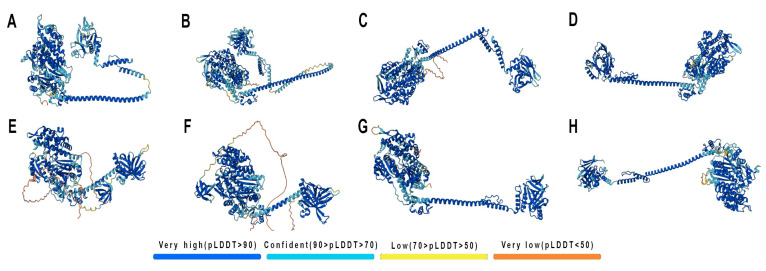
The 3D structures of human myosin class I proteins. The color scale represents the predicted local distance difference test (pLDDT) reflecting the accuracy of the predicted 3D structures. Regions predicted with the highest confidence level (100 > pLDDT > 90) are shown in blue. Regions with 90 > pLDDT > 70 are shown in cyan. Regions with 70 > pLDDT > 50 are shown in yellow. Regions with low prediction confidence (pLDDT < 50) are shown in orange. (**A**) myosin IA; (**B**) myosin IB; (**C**) myosin IC; (**D**) myosin ID; (**E**) myosin IE; (**F**) myosin IF; (**G**) myosin IG; (**H**) myosin IH.

The three-dimensional myosin I models were built using the AlphaFold Server (Figure 1), and they showed a high mean predicted accuracy (predicted local distance difference test, pLDDT > 85). All analyzed proteins maintained a conserved architecture comprising three structural domains: N-terminal motor domain predominantly composed of α-helices, central neck domain represented by a single α-helix, and a more compact C-terminal tail domain. However, myosins IC, IE, and IF have extensive regions with low prediction confidence (pLDDT < 50). These regions may correspond to intrinsically disordered regions (IDRs), distinguished by their flexibility and dynamics, which lack a single stable three-dimensional conformation and acquire one only upon binding to a partner. Myosin IC has the N-terminal unstructured region that differentiates it from other long-necked myosins, IA and IB. Myosins ID, IG, and IH are well-predicted and very similar in structure with medium-length neck domains. The short-necked myosins, IE and IF, both have large C-terminal unstructured regions. These may influence the functional interactions of these proteins.

The detailed characterization of the myosins’ surface properties (Supplementary Figures S1 and S2) demonstrated the predominance of hydrophilic and amphipathic regions over the hydrophobic ones. This is consistent with myosin function in the cytosol; however, the size and distribution of hydrophobic regions may influence specific membrane interactions of these proteins. An investigation of the electrostatic potential revealed that the motor domain of class I myosins bears neutral or weakly negative charge. The tail domains contain positively charged areas of various sizes, and the neck domains are positively charged in all myosin I proteins. Thus, class I myosins show structural similarity, however, the differences in neck domain length, unstructured regions, and the localization of charged areas may influence their selective functions.

### 2.2. In Silico Analysis of MYO1 Gene Expression in a Human Cell Line Dataset

**Figure 2:**
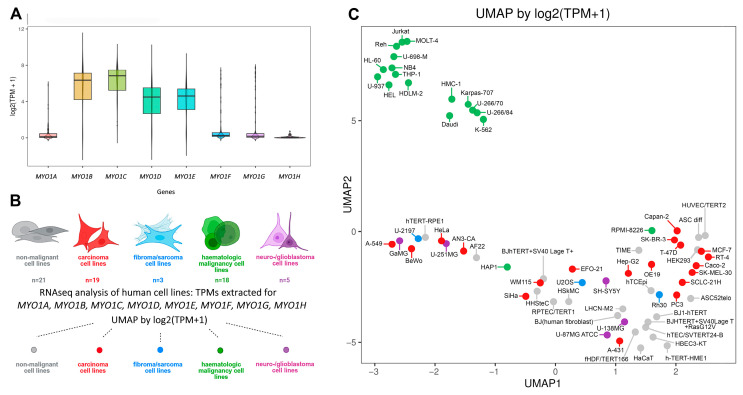
Myosin class I genes analyzed in silico on a dataset of 66 human cell lines. (**A**) Distribution of *MYO1A*, *MYO1B*, *MYO1C*, *MYO1D*, *MYO1E*, *MYO1F*, *MYO1G*, and *MYO1H* transcripts in the cell line array. (**B**) The infographics based on the manual cell line annotation (Created in BioRender. Saidova, A. (2026) https://BioRender.com/xigiyeu (accessed on 28 November 2025)). The cell lines in the dataset were subdivided into: non-malignant cell lines of different origin (gray), malignant carcinomas (red), malignant fibromas/sarcomas (blue), haematologic malignancies (green); and malignant neuroblastomas/glioblastomas (purple). The colors in B correspond to the dot color in C for plotted cell lines. (**C**) UMAP analysis of the 66 cell lines. Each cell line is plotted as a dot. The color of the dot corresponds to the cell line annotation shown in B.

To investigate the transcriptomic differences in *MYO1* genes in different cell and tissue types, we re-analyzed the available RNA-seq database of human cell lines. Upon manual annotation of the dataset, three cell lines were excluded from analysis (two of the cell lines were germline tumors—a distinct type of tumor underrepresented in the current dataset; one cell line had questionable origin). The final dataset comprised 66 human malignant and non-malignant cell lines. The log2-transformed TPM (transcripts per million) values for each human class I myosin (*MYO1A*, *MYO1B*, *MYO1C*, *MYO1D*, *MYO1E*, *MYO1F*, *MYO1G*, and *MYO1H*) were plotted to assess the transcript frequency in the array of human cell lines (Figure 2A). Subsequently, four human myosins (*MYO1B*, *MYO1C*, *MYO1D*, and *MYO1E*) had high transcript frequency in the majority of specimens; the other four (*MYO1A*, *MYO1F*, *MYO1G*, and *MYO1H*) had considerably lower transcript frequency. The differences in myosin I gene expression levels between individual cell lines are presented in Supplementary Figure S3.

Upon manual annotation (see Supplementary Table S2), the cell lines were subdivided into non-malignant cell lines of different origin (n = 21), malignant carcinomas (n = 19), malignant fibromas/sarcomas (n = 3), haematologic malignancies (n = 18), and malignant neuroblastomas/glioblastomas (n = 5) (Figure 2B). To verify if the expression pattern of MYO1 genes allows to distinguish any of these groups from others, the uniform manifold approximation and projection (UMAP) analysis was performed; the results are presented in Figure 2C. Haematologic malignancies were the only group distinguished from the others by the expression of eight *MYO1* genes: only two relevant cell lines (HAP-1 and RPMI-8226) failed to fall within this group on a plot. Notably, using whole-transcript UMAP only helped to add the RPMI-8226 cell line to this group and helped to resolve other groups but a little better (see Supplementary Figure S4).

**Figure 3:**
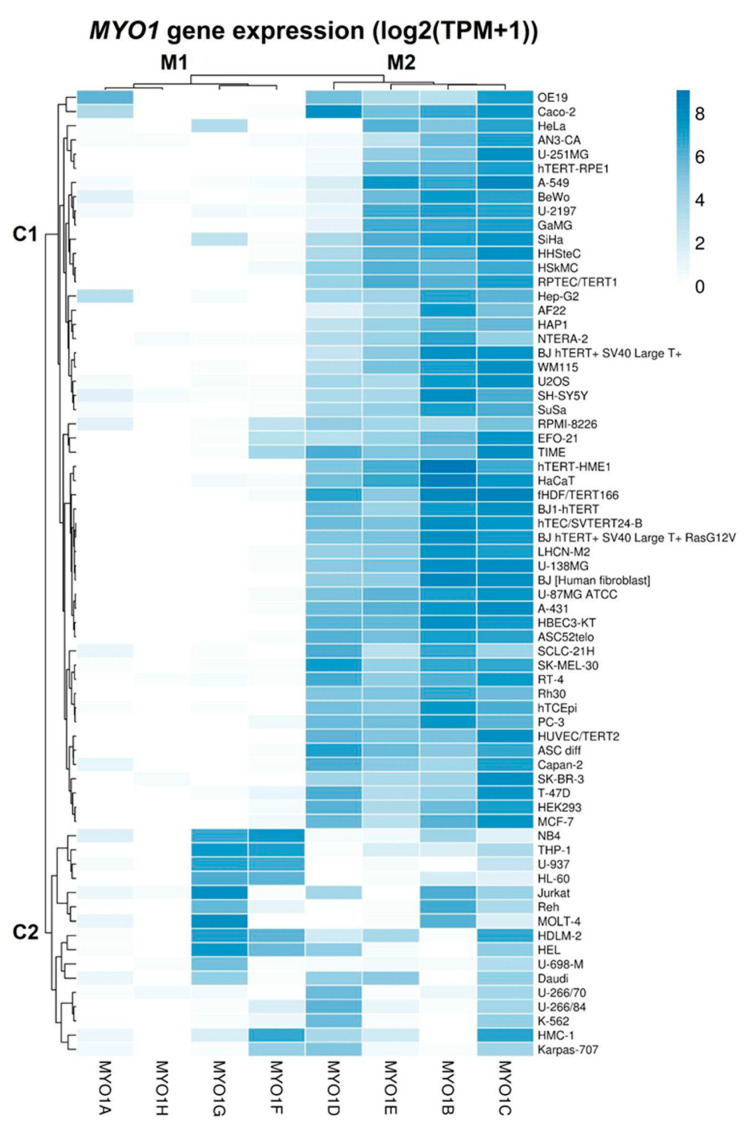
The Ward clusterization analysis of myosin I transcription in 66 cell lines (the linear distance in the cluster plot represents the Euclidean distance between specimens). Cluster C1 contains cell lines unspecified by group. Cluster C2 contains cell lines of haematologic malignancies. *MYO1* genes are clustered into low frequency (M1) and high frequency (M2) groups with moderate and high expression respectively.

To define the expression patterns for individual *MYO1* genes in the analyzed cell lines, we plotted the log2-transformed TPM values for the eight *MYO1* genes as a heatmap for all cell lines and clustered the data through the Ward method (the lines represent the Euclidean distances as a measure of similarity between specimens). The results are presented in Figure 3.

The *MYO1* genes clustered into the low frequency myosin (M1) and high frequency myosin (M2) transcript groups that contained the *MYO1A*, *MYO1F*, *MYO1G*, and *MYO1H* and the *MYO1B*, *MYO1C*, *MYO1D*, and *MYO1E* genes respectively. The cell lines were primarily clustered into two groups. Cluster C2 contained all haematologic malignancy cell lines except HAP-1 and RPMI-8226. Cluster C1 contained all other cell lines that did not show any enrichment by type in the following subclusters. This supports the data of the UMAP analysis. The distinctive features of the C2 cluster are the depletion of high frequency M2 myosin transcripts and the high expression of *MYO1G* and *MYO1F* of the low frequency M2 myosin transcripts compared to C1. Thus, the clusterization data support the UMAP analysis and expand on the specific *MYO1* expression signature in the group of haematologic malignancy cell lines.

### 2.3. In Silico Analysis of MYO1 Gene Expression in Human Cell Lines of Different Origin and Malignancy Status

To analyze *MYO1* genes’ expression in human malignancies and normal tissues of different origin more precisely, we subdivided the cell line set further: by tissue type and malignancy status (Supplementary Table S2). The dataset contained a sufficient amount of malignant and non-malignant cell lines derived from epithelial tissue and connective tissue. The neurological tumors were excluded from further analysis as the group was underrepresented and did not contain any normal neurological counterparts.

**Figure 4:**
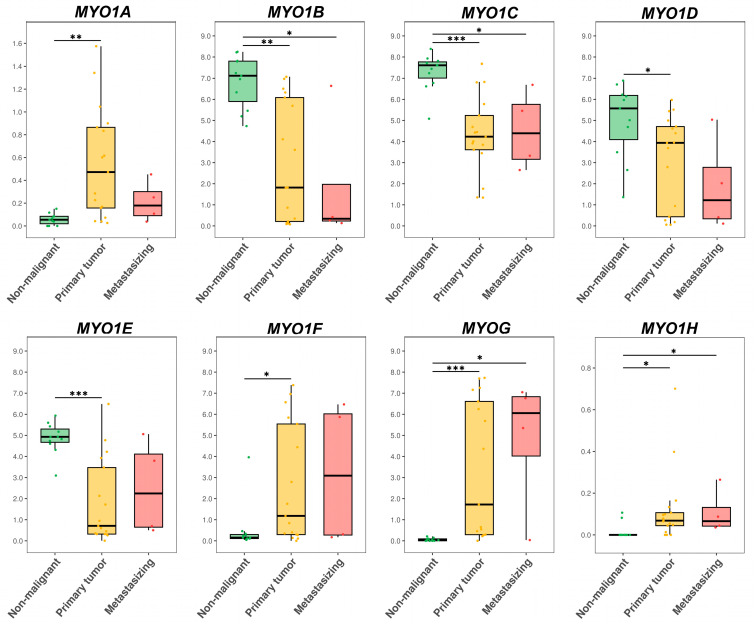
The comparison of *MYO1A*, *MYO1B*, *MYO1C*, *MYO1D*, *MYO1E*, *MYO1F*, *MYO1G*, and *MYO1H* expression levels in non-malignant and malignant (primary and metastasizing) connective tissue cell lines. The log2(TPM + 1) values are plotted for each gene. Groups of 11 non-malignant cell lines (fibroblasts, endothelium, MSCs, etc.) of 17 primary tumors (fibroma/sarcoma + haematologic malignancies) and 4 metastasizing tumors (fibroma/sarcoma + haematologic malignancies) were compared using the Mann–Whitney U test with Benjamini–Hochberg (BH) correction. The levels of significance: *—*p* < 0.05, **—*p* < 0.01, and ***—*p* < 0.001.

The connective tissue group contained 11 non-malignant cell lines (fibroblasts, endothelium, MSCs, etc.) and 21 malignant cell lines (fibroma/sarcoma + haematologic malignancies). The malignant cell lines were further divided by migratory potential into a group of primary tumors (for cells derived from primary tumor, n = 17) and a metastasizing group (for cells derived from metastases, either distant or lymph node, n = 4). The log2-transformed TPM values of each *MYO1* gene were compared between these three groups using the Mann–Whitney U test with Benjamini–Hochberg (BH) correction. The results are presented in Figure 4.

A significant decrease was observed for *MYO1B* and *MYO1C* in the primary and metastasizing groups compared to the non-malignant group. Also, there was a significant decrease in *MYO1D* and *MYO1E* expression in the primary tumor group compared to the non-malignant group. A significant increase was observed for *MYO1G* and *MYO1H* in the primary and metastasizing groups compared to the non-malignant group. Also, there was a significant increase in *MYO1A* and *MYO1F* expression in the primary group compared to the non-malignant group. Thus, significant shifts were observed for all *MYO1* genes.

**Figure 5:**
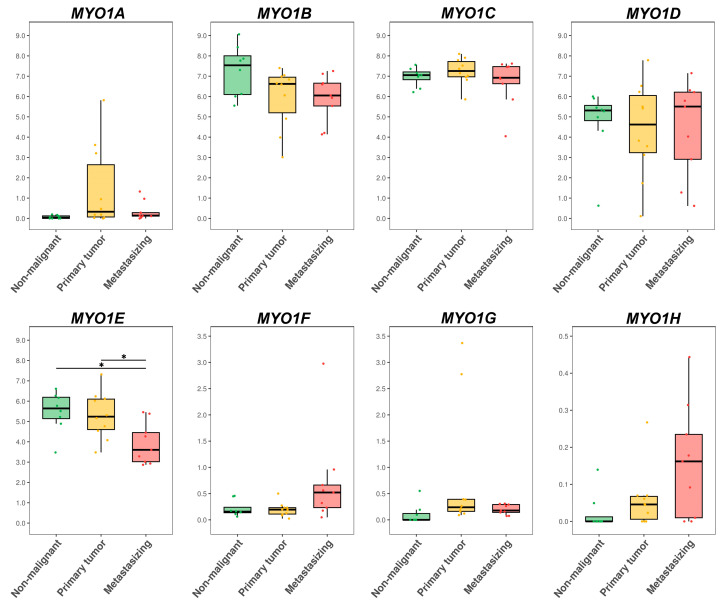
The comparison of *MYO1A*, *MYO1B*, *MYO1C*, *MYO1D*, *MYO1E*, *MYO1F*, *MYO1G*, and *MYO1H* expression levels in non-malignant and malignant (primary and metastasizing) epithelial tissue cell lines. The log2(TPM + 1) values are plotted for each gene. Groups of 8 non-malignant epithelial cell lines, 10 primary carcinomas, and 9 metastasizing carcinomas were compared using the Mann–Whitney U test with Benjamini–Hochberg (BH) correction. The levels of significance: *—*p* < 0.05.

The epithelial tissue group contained 8 non-malignant epithelial cell lines of different origins and 19 carcinoma cell lines, further subdivided by migratory potential into a primary group (for cells derived from primary tumor, n = 10) and a metastasizing group (for cells derived from metastases, either distant or lymph node, n = 9). The log2-transformed TPM values of each *MYO1* gene were compared between these three groups using the Mann–Whitney U test with Benjamini–Hochberg (BH) correction. The results are presented in Figure 5. The only significant difference was observed for *MYO1E*. It was decreased in the metastasizing group compared to both the primary and non-malignant groups.

### 2.4. Analysis of MYO1 Gene Expression in Cultured Cell Lines Using the Developed qPCR System

The qPCR system was developed to assess the expression levels of all class I myosins (*MYO1* genes) simultaneously in a simple and cost-effective assay. To test the system, a total of 35 continuous human cell lines were obtained from our own repository and donated from the repositories of the V.N. Orekhovich Institute of Biomedical Chemistry and the N.N. Blokhin National Medical Research Center of Oncology. Similarly to the RNA-seq data analysis, the annotation was made manually for cell lines used in the experiments (see Supplementary Table S3).

The primers were designed in accordance with standard recommendations [51] so that they would detect all known isoforms of *MYO1* genes for total representation analysis. The sequences for the primers are presented in Supplementary Table S4. Primers were tested on our sample set in the SYBR Green I qPCR format: the amplification was verified, and the melting temperature for each product was assessed through the melting curve analysis and added to Supplementary Table S4. The MYO1C gene was expressed in all specimens, although it was quite decreased in haematopoietic cell lines. The low frequency genes *MYO1H* and *MYO1F* failed to amplify in our sample set. All other genes, *MYO1A*, *MYO1B*, *MYO1D*, *MYO1E* and *MYO1G*, were expressed in a number of cell lines and were absent in the others. A single product of expected length was confirmed for each myosin I through agarose electrophoresis, and the absence of product was confirmed likewise. The representative results are shown in Supplementary Figures S5 and S6.

Two reference genes, namely *YWHAZ* and *UBC*, were chosen for data normalization. Their amplification in the cell lines of choice was primarily confirmed through the SYBR Green I qPCR. However, TaqMan probes were then designed for *MYO1* genes and the reference genes to bring the qPCR system to the uniform multiplexed TaqMan format. The probes with either VIC or ROX labeling are presented in Supplementary Table S4. In silico analysis, it was confirmed that the primers and probes in the study could be multiplexed in pairs. The genes were paired as follows: *YWHAZ + MYO1B*, *UBC + MYO1C*, *MYO1D + MYO1G*, *MYOF + MYOE*, and *MYOA + MYOH*; Supplementary Figure S7 shows representative multiplexed reactions. The final qPCR experiment was run in multiplexed reactions, and the relative expression values (delta Ct) for *MYO1* genes were normalized through the normalization factor (the geometric mean of *YWHAZ* and *UBC* relative expression in the specimen). The normalized relative quantities (NRQ) for *MYO1* genes are provided in Supplementary Table S5.

**Figure 6:**
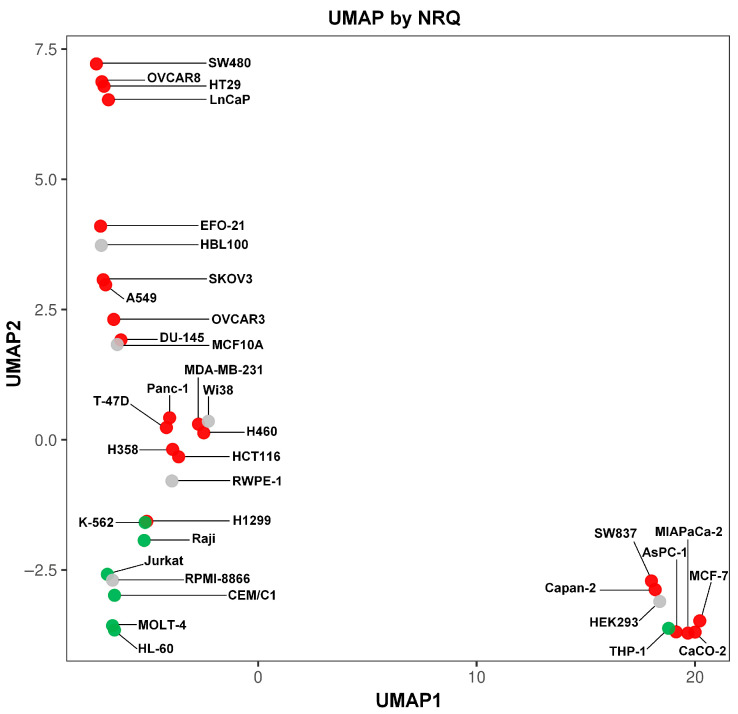
UMAP analysis of the qPCR data on 35 human cell lines. Each cell line is plotted as a dot, the choice of color for the dots is similar to Figure 1C and corresponds to the cell line annotation: non-malignant cell lines of different origin (gray), malignant carcinomas (red), and haematologic malignancies (green).

**Figure 7:**
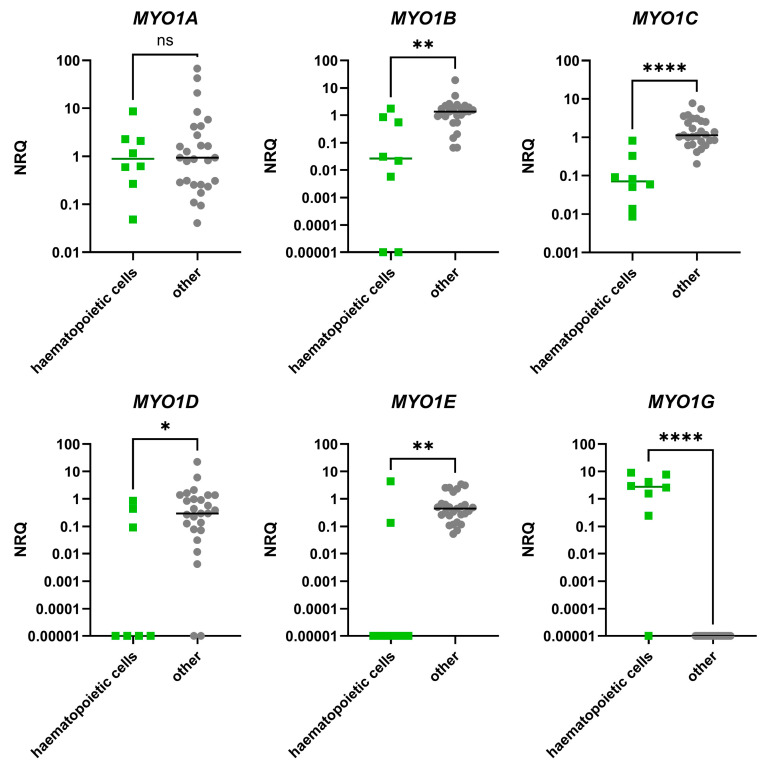
The qPCR data on 35 human cell lines (haematopoietic (n = 8) and non-hematopoietic (other, n = 27) cell lines presented as normalized relative quantities (NRQs) and compared using Mann–Whitney test. The levels of significance: *—*p* < 0.05, **—*p* < 0.01, ****—*p* < 0.0001, and ns—non-significant.

The UMAP analysis was run on the qPCR data, and although it showed some differences from the RNA-seq data, it allowed us to visualize haematologic malignancies together (Figure 6). The haematologic malignancy cell lines, except THP-1, were plotted together with the normal haematopoietic B-cell line RPMI-8866. The distinction from other cell lines was less apparent than in the UMAP analysis of RNA-seq data; however, the differences implied a specific pattern of *MYO1* expression. To directly test this hypothesis and compare *MYO1* expression between haematopoietic and non-haematopoietic cell lines, we plotted them against each other and compared the NRQs (Figure 7).

The clusterization of log10-transformed NRQs through the Ward method also revealed a subcluster enriched in haematopoietic cell lines (cluster C2_1, Supplementary Figure S8). Taken together, the qPCR data suggest that haematopoietic cell lines are distinct from others and are characterized by decreased expression of *MYO1B*, *MYO1C*, *MYO1D*, and *MYO1E* and an increase in *MYO1G*. These data are in accordance with the RNA-seq analysis.

## 3. Discussion

Many pathologies are associated with cytoskeleton reorganization and malfunction. Class I myosins are a group of cytoskeletal proteins that currently attract a lot of attention in the context of health and disease [52,53]. Their studies, however, are complicated by the large number of members in this class: eight related proteins (as shown in Figure 1) are currently known in mammals, including humans. Some of them also have isoforms, which further complicates their representation patterns and possibly the range of their functions. While the general domain structure is shared by all class I myosins, their domain size, the localization and size of their unstructured regions, and the distribution of hydrophilic/hydrophobic and charged surface areas are different, which may direct their selective binding preferences, interactions and roles in cells. The specific properties of class I myosins are now being addressed in structural studies and interaction modeling [54,55]; however, much is yet to be done to comprehensively describe their diversity and functions in normal and pathological conditions.

The existing data suggest the tissue-specific distribution of different class I myosins, although their finalized expression patterns in human cells are still lacking. Here we used an array of popular model cell lines to simultaneously describe the transcription patterns of all human *MYO1* genes, which has not been done before. Known human cell lines were chosen for analysis, as they exhibit less intrinsic heterogeneity than ex vivo tissue and present common and well-described in vitro models in different research areas. The RNA-seq dataset (GSE240542) was chosen as it contains both malignant cell lines and non-malignant cell lines for comparison [56]. The initial assessment of the RNA-seq revealed four abundantly expressed *MYO1* genes (*MYO1B*, *MYO1C*, *MYO1D*, and *MYO1E*) and four moderately expressed genes (*MYO1A*, *MYO1F*, *MYO1G*, and *MYO1H*), with *MYO1H* being ultimately rare. As direct comparison of all *MYO1* genes in human cells has not been implicated so far, these data may be of interest in further functional studies and biomarker research. The less frequently expressed genes may encode proteins with restricted roles in cells, and their expression may be associated with some functional changes.

We attempted to find any specific expression patterns of the eight *MYO1* genes that would distinguish cell lines by type, as was done using the full omics approach [56,57,58]. Here, we showed that the expression of eight *MYO1* genes was sufficient to define a group of cell lines representing haematologic malignancies from all other model cells. Their segregation through the UMAP analysis implies extensive changes in the expression profile. We went on to show that the unique profile was associated with decreased expression of all abundantly expressed *MYO1* genes (*MYO1B*, *MYO1C*, *MYO1D*, and *MYO1E*) and elevated expression of the rare genes—*MYO1G* and *MYO1F*. This distinctive pattern was uniform in the broad range of haematologic malignancy cell models in the dataset. To date, myosin IG is primarily associated with human blood disorders as a biomarker of childhood acute lymphoblastic leukemia [48,49]. Our data show that assessing the whole pattern of *MYO1* expression would potentially uncover more differences and create a more detailed picture of pathological changes. Also, other haematologic malignancies could be associated with *MYO1* expression shifts which requires further investigation.

The pattern of *MYO1* expression was so distinctive in the group of haematologic malignancies that it has possibly affected the analysis of *MYO1* genes in connective tissue cell lines. To account for possible tissue specificity, we analyzed all cell lines of connective tissue origin separately to determine the tumor-associated *MYO1* expression shifts. Comparing the primary and metastasizing tumor cell lines (including haematopoietic tumors) to the normal connective tissue cells yielded significant expression differences for all *MYO1* genes. However, the absence of direct haematopoietic non-malignant counterparts in the dataset does not allow us to determine the input of pan-leukocytic or tumor-specific expression to the observed *MYO1* pattern. The data obtained on non-malignant mouse models suggest that *MYO1G* expression may be leukocyte-specific. It is expressed in non-malignant murine T- and B-lymphocytes and regulates cytoskeleton plasticity, cell polarization and migration, and exocytosis and endocytosis [59,60,61,62]. If the same is true for human cells, any bulk analysis of *MYO1* genes in any tissue, including tumors, will be sensitive to the amount of immune cell infiltrates, and thus will need cautious interpretation. To date, a direct comparison of the whole *MYO1* expression pattern in malignant and non-malignant human leukocyte populations is still to be done.

In contrast to connective tissue, epithelial cell line analysis did not reveal a lot of differences. Carcinoma cell lines failed to clusterize as a separate entity and did not exhibit any specific *MYO1* pattern. Comparing primary and metastasizing carcinomas and normal cells of epithelial origin revealed only one systemic alteration—a decrease in *MYO1E* expression in metastasizing carcinomas compared to primary carcinomas and non-malignant epithelial cell lines. This may reflect an important aspect of their biology that requires further investigation. Regarding the myosin IE function in maintaining lamellipodia adhesion and regulating cell contacts via FAK kinase [63,64], its loss could enhance the migratory potential of epithelial tumors. Other *MYO1* expression changes that may occur in cancer appear less extensive and require targeted approach using human biopsies and relevant controls.

We designed a qPCR system to compare the expression *MYO1* genes in a simple and cost-effective way. Clustering qPCR data also allowed us to segregate haematopoietic cell lines from others. The only non-malignant haematopoietic B-cell line in our set, RPMI-8866, was plotted close to haematologic malignancies, thus supporting the notion that the revealed *MYO1* pattern was leukocyte-specific. Direct comparison showed significantly elevated *MYO1G* and significantly decreased *MYO1B*, *MYO1C*, *MYO1D*, and *MYO1E* in haematopoietic vs. non-haematopoietic cell lines. All these changes were consistent with the RNA-seq analysis. However, unlike the RNA-seq, qPCR results indicated the absence of *MYO1F* in haematopoietic cell lines in our dataset. As myosin 1F was previously associated with activation of various immune cells in different experimental environments [30,31,65,66,67], such discrepancy can be explained by different activation states of cultured haematopoietic cell lines in the sample sets. This implies the use of full *MYO1* profiling to study such complex processes as immune activation, since the changes may affect the underexpressed *MYO1* genes.

Taken together, our data support the notion of cell-type specific expression of *MYO1* genes and allow to segregate haematopoietic cell lines based on the full *MYO1* expression pattern. The major limitation of the current study is using cell lines cultured in vitro. Translating these results to human ex vivo specimens may be associated with difficulties and require further studies on biopsies and single-cell profiling. However, the cell lines present the most popular and available models widely used in biology and biomedicine, and a detailed *MYO1* profile sets a baseline for further functional studies and biomarker research.

## 4. Materials and Methods

### 4.1. In Silico Analysis of Protein Structures

The UniProt database was used to predict the protein structures (UniProt IDs: Q9UBC5 (Myosin IA); O43795 (Myosin IB); O00159 (Myosin IC); O94832 (Myosin ID); Q12965 (Myosin IE); O00160 (Myosin IF); B0I1T2 (Myosin IG); and Q8N1T3 (Myosin IH)). Three-dimensional (3D) models of human class I myosins were built using the AlphaFold Server algorithm (https://alphafoldserver.com (accessed on 23 January 2026)). The final models were visualized with a pLDDT (predicted local distance difference test) color scale. The yielded structures had a high mean predicted accuracy (pLDDT > 85).

The analysis of the surface and charge of the predicted 3D myosin structures was performed using UCSF Chimera v.1.17.3. Surface hydrophobicity visualization was performed via Presets: Interactive 3 (hydrophobicity surface). Charge visualization was performed using Tools -> Surface/Binding Analysis -> Coulombic Surface Coloring.

### 4.2. Meta-Analysis of RNA-seq Datasets

The published bulk RNA-seq data from human cultured cell lines were re-analyzed to identify the tissue and tumor specificity of *MYO1* genes. The RNA-seq dataset reported by Jin et al. [56] was chosen (GSE240542) as it uses sufficient experimental replicates and contains a large amount of non-malignant cell lines. Raw RNA-seq data were retrieved from the NCBI Sequence Read Archive (SRA) using the SRA Toolkit (v3.2.1) and converted into the FASTQ format. Quality filtering and adapter removal were performed using fastp (v1.0.1). Transcript expression was quantified by aligning reads to the human reference genome GRCh38.p14 using the GENCODE Release 47 annotation. Read alignment was performed with STAR (v2.7.10). Strand specificity of each library was evaluated using infer_experiment.py from the RSeQC package (v5.0.4). Gene-level read counts were obtained using featureCounts (v2.1.1). The annotation of the cell lines used in the in silico study was manual: cell lines were stratified based on the tissue type, malignancy/non-malignancy, and their putative metastatic properties. The summary is presented in Supplementary Table S2. A total of 66 cell lines was included in the analysis.

### 4.3. Cell Lines

The 35 continuous human cell lines of different origins widely used as consensus in vitro models were included in the qPCR analysis of *MYO1* genes. Cell lines were obtained from the School of Biology of M.V. Lomonosov Moscow State University, V.N. Orekhovich Institute of Biomedical Chemistry, and N.N. Blokhin National Medical Research Center of Oncology. The descriptions of the cell lines used for qPCR are summarized in Supplementary Table S3. The cells were stored at −80 °C, then thawed and maintained for qPCR experiments. All cell lines were cultured at 37 °C in a humid atmosphere in the presence of 5% CO_2_.

The eight suspension cell lines, including B-cells (Raji, RPMI-8866), T-cells (Jurkat, MOLT-4, CEM/C1), monocytes (THP-1), and myeloid cells (HL-60, K-562), were maintained in the RPMI-1640 medium with 10% FBS, 2 mM L-glutamine, and 100 U/mL penicillin/streptomycin (all reagents were supplied by PanEco, Moscow, Russia).

Adherent Cell Lines Were Maintained as Follows:

The 18 cell lines corresponding to colon carcinoma (HT29, CaCO-2, HCT-116, SW480, and SW837), lung carcinoma (A549, H1299, H460, and H358), prostatic carcinoma (DU-145), ovarian carcinoma (SKOV3), mammary carcinoma (MDA-MB-231 and MCF-7), pancreatic carcinoma (PANC-1, Capan-2, and MIAPaCa-2) and non-malignant cells (Wi38 and HEK293) were cultured in DMEM with 10% FBS, 2 mM L-glutamine, and 100 U/mL penicillin/streptomycin (all reagents were supplied by PanEco, Moscow, Russia).

The HBL-100 cell line was cultured in the DMEM/F12 medium with 10% FBS, 2 mM L-glutamine, and 100 U/mL penicillin/streptomycin (all reagents were supplied by PanEco, Moscow, Russia).

The six cell lines corresponding to ovarian carcinoma (EFO-21, OVCAR-3, and OVCAR-8), prostatic carcinoma (LnCaP), pancreatic carcinoma (AsPC1), and mammary carcinoma (T-47D) were cultured RPMI-1640 with 15% FBS, 2 mM L-glutamine, and 100 U/mL penicillin/streptomycin (all reagents were supplied by PanEco, Moscow, Russia).

The MCF10A cell line was cultured in the DMEM/F12 medium (Gibco, Thermo Fisher Scientific, Waltham, MA, USA) supplemented with 5% horse serum (Hyclone, Cytiva, South Logan, UT, USA), 100 ng/mL cholera toxin (Sigma-Aldrich, St. Louis, MO, USA), 20 ng/mL epidermal growth factor (Sigma-Aldrich, St. Louis, MO, USA), 0.01 mg/mL insulin (PanEco, Moscow, Russia), 500 ng/mL hydrocortisone (Sigma-Aldrich, St. Louis, MO, USA), and 100 U/mL penicillin/streptomycin (PanEco, Moscow, Russia).

The RWPE-1 cell line was cultured in Keratinocyte-SFM (Gibco, Thermo Fisher Scientific, Waltham, MA, USA) with 50 ug/mL BPE (Gibco, Thermo Fisher Scientific, Waltham, MA, USA), 5 ng/mL EGF (Gibco, Thermo Fisher Scientific, Waltham, MA, USA), and 100 U/mL penicillin/streptomycin (PanEco, Moscow, Russia).

### 4.4. RNA and cDNA

Nucleic acids were isolated from 100 μL of each cell suspension (containing 400,000–500,000 cells) using the RIBO-prep kit (AmpliSens, Moscow, Russia) according to the manufacturer’s protocol. After digestion with DNase E (Evrogen, Moscow, Russia) according to the manufacturer’s instruction, the RNA was re-isolated using the RIBO-prep kit. The resulting RNA was stored at −70 °C.

Reverse transcription with 0.8–1.0 ug RNA per reaction was performed using the MMLV Reverse Transcription kit by Evrogen (Moscow, Russia) according to the manufacturer’s protocol. The resulting cDNA was used for real-time qPCR.

### 4.5. Primer and Probe Design

Sets of primers and probes for each of the eight human *MYO1* genes were designed in accordance with standard recommendations [51]. Oligo Analyzer software version 1.0.3 (OligoSoftware, Kuopio, Finland) was used to assess the melting temperatures of the oligonucleotides and the absence of stable secondary structures. The absence of non-specific amplification was verified using the online Primer-BLAST instrument (https://www.ncbi.nlm.nih.gov/tools/primer-blast/ (accessed on 8 August 2025)). All oligonucleotides were synthesized commercially by DNA Synthesis (Moscow, Russia). The properties of the primers and probes, including the gene IDs, sequences, and melting temperatures are listed in Supplementary Table S4. As the primers were aligned to have similar melting temperatures (~60 °C), the annealing temperature in all reactions was set to 58 °C.

### 4.6. Real-Time qPCR

All primers and probes for the experiments were produced by Syntol (Moscow, Russia).

The human *MYO1* genes (*MYOIA*, *MYOIB*, *MYOIC*, *MYOID*, *MYOIE*, *MYOIF*, *MYOIG*, and *MYOIH*) were first tested using SYBRGreen I qPCR. The reagents were purchased from Syntol, Russia, as a SYBRGreen I qPCR detection kit and used in accordance with the manufacturer’s instructions. The reaction protocol included denaturation (95 °C, 3 min) followed by 39 amplification cycles (95 °C, 15 s; 58 °C, 30 s; and 72 °C, 40 s). Samples were processed in triplicate using a CFX96 Touch cycler with CFX-Manager 3.1 software (Bio-Rad, Hercules, CA, USA). The *UBC* and *YWHAZ* genes were used for data normalization. To pool the data from different plates, the same cDNA sample was used as a calibrator.

For further data validation, a multiplexed TaqMan qPCR system was developed using VIC- or ROX-labeled fluorescent probes. In the multiplexed system, the genes were paired as follows: *YWHAZ + MYO1B*, *UBC + MYO1C*, *MYO1D + MYO1G*, *MYOF + MYOE*, and *MYOA + MYOH*. The qPCR reactions were run using a qPCRmix-HS (Evrogen, Russia); the reaction protocol included denaturation (95 °C, 5 min) followed by 39 amplification cycles (95 °C, 15 s; 58 °C, 30 s; and 72 °C, 40 s). Similar to the SYBRGreen I protocol, samples were processed in triplicate, the *UBC* and *YWHAZ* genes were used for data normalization, and the same cDNA sample was used as a calibrator to pool data from different plates. The relative expression values (delta Ct) for *MYO1* genes were normalized through the normalization factor (the geometric mean of *YWHAZ* and *UBC* relative expression in the specimen). The qPCR data was presented as normalized relative quantities (NRQ).

### 4.7. End-Point PCR

Horizontal agarose gel electrophoresis was used to qualitatively verify the qPCR data. Amplification was run without fluorescence detection using the same cycle protocol as in qPCR; 5 uL aliquots of the PCR products were mixed with 2 uL of the leading dye OrangeG (Sigma-Aldrich, St. Louis, MO, USA). The mixtures were applied to the wells of a 2% agarose (Merck, Darmstadt, Germany) gel prepared in Tris-acetate buffer with EDTA (TAE 1X). Ethidium bromide (5 uL of 10 mg/mL stock, Merck, Darmstadt, Germany) was added to the gel to allow subsequent band detection under UV light. A ready-to-use DNA Ladder kit (Evrogen, Moscow, Russia) was used as a molecular weight marker. Electrophoresis was performed in a horizontal electrophoresis chamber (Helicon, Moscow, Russia) at a constant voltage of 117 V for 30–40 min in TAE buffer (1X).

### 4.8. Data Analysis

Data analysis and plotting for TPM (transcripts per million) retrieved from RNA-seq and NRQ (normalized relative quantities) retrieved from qPCR were done in R. Data visualization was performed using uniform manifold approximation, projection (UMAP) on log_2_(TPM + 1) expression values, and NRQ expression values. UMAP embedding was generated with the umap R package (v0.2.10.0) using default parameters. Boxplots and other visualizations were produced with ggplot2 (v3.5.2).

Pairwise group comparisons were conducted using the Wilcoxon rank-sum test (Mann–Whitney U test) implemented in the base R package stats (v4.3.2). *p*-values were adjusted for multiple testing using the Benjamini–Hochberg procedure via the p.adjust function (stats). Data manipulation and table construction were performed using the dplyr (v1.1.4), purrr (v1.0.4), and tibble (v3.3.0) packages in R.

All codes to reproduce transcriptomic meta-analysis are available at: https://github.com/ArtyomTyukaev/MYO1-RNAseq-analysis (accessed on 1 December 2025).

## Data Availability

The data presented in this study are available in the article and in the Supplementary Materials. All codes to reproduce transcriptomic meta-analysis are available on GitHub at https://github.com/ArtyomTyukaev/MYO1-RNAseq-analysis (accessed on 11 December 2025).

## Supplementary Materials

The following supporting information can be downloaded at: https://www.mdpi.com/article/10.3390/ijms27041777/s1.
