# Supplementary material for: Myosin Class I Genes Follow a Cell Type-Specific Transcription Pattern in Human Haematopoietic Cell Lines
Source: Int J Mol Sci. 2026 Feb 12;27(4):1777. doi: 10.3390/ijms27041777 (PMC12940969; doi:10.3390/ijms27041777)
Supplement: Supplementary file 1 [file ijms-27-01777-s001.zip › Supplementary Data/Supplementary Figures_Myosin class I.pdf]

Myosin class I genes follow a cell type-specific transcription pattern in human haematopoietic cell lines
Supplementary Figures

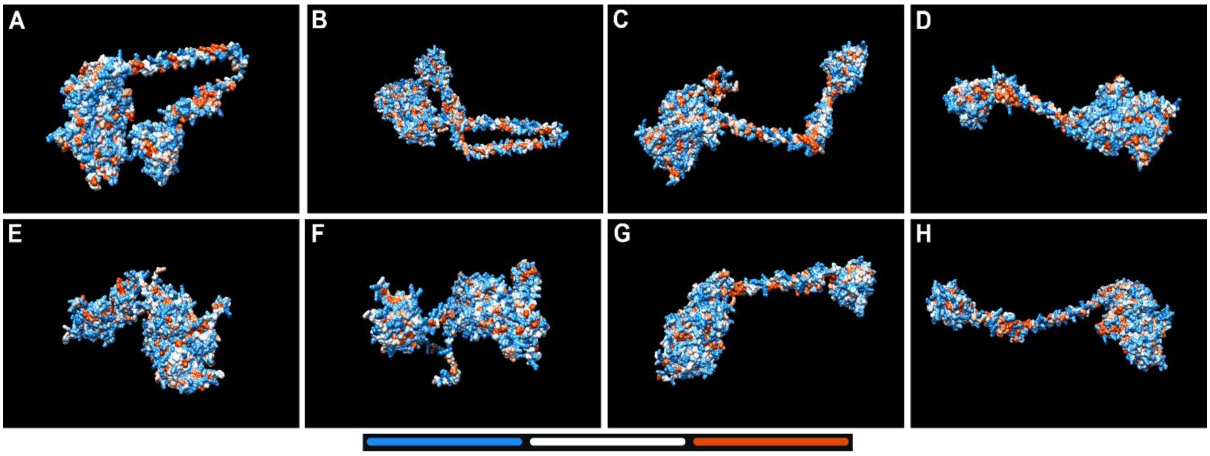

Supplementary Figure S1. The 3D structures of myosin class I proteins with predicted hydrophobic potential. The potentially hydrophilic (blue), hydrophobic (red), and amphipathic (white) regions are color-mapped to the surface of class I myosin proteins. A – myosin IA; B – myosin IB; C – myosin IC; D – myosin ID; E – myosin IE; F – myosin IF; G – myosin IG; H – myosin IH.

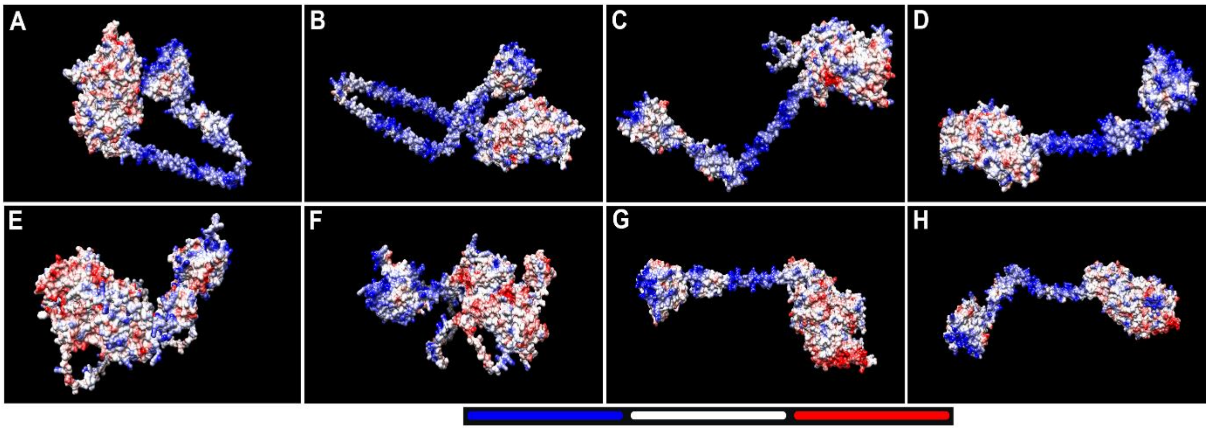

Supplementary Figure S2. The 3D structures of myosin class I proteins with predicted surface charge. The positively (blue), neutrally (white), and negatively (red) charged regions are color-mapped to the surface of class I myosin proteins. A – myosin IA; B – myosin IB; C – myosin IC; D – myosin ID; E – myosin IE; F – myosin IF; G – myosin IG; H – myosin IH.

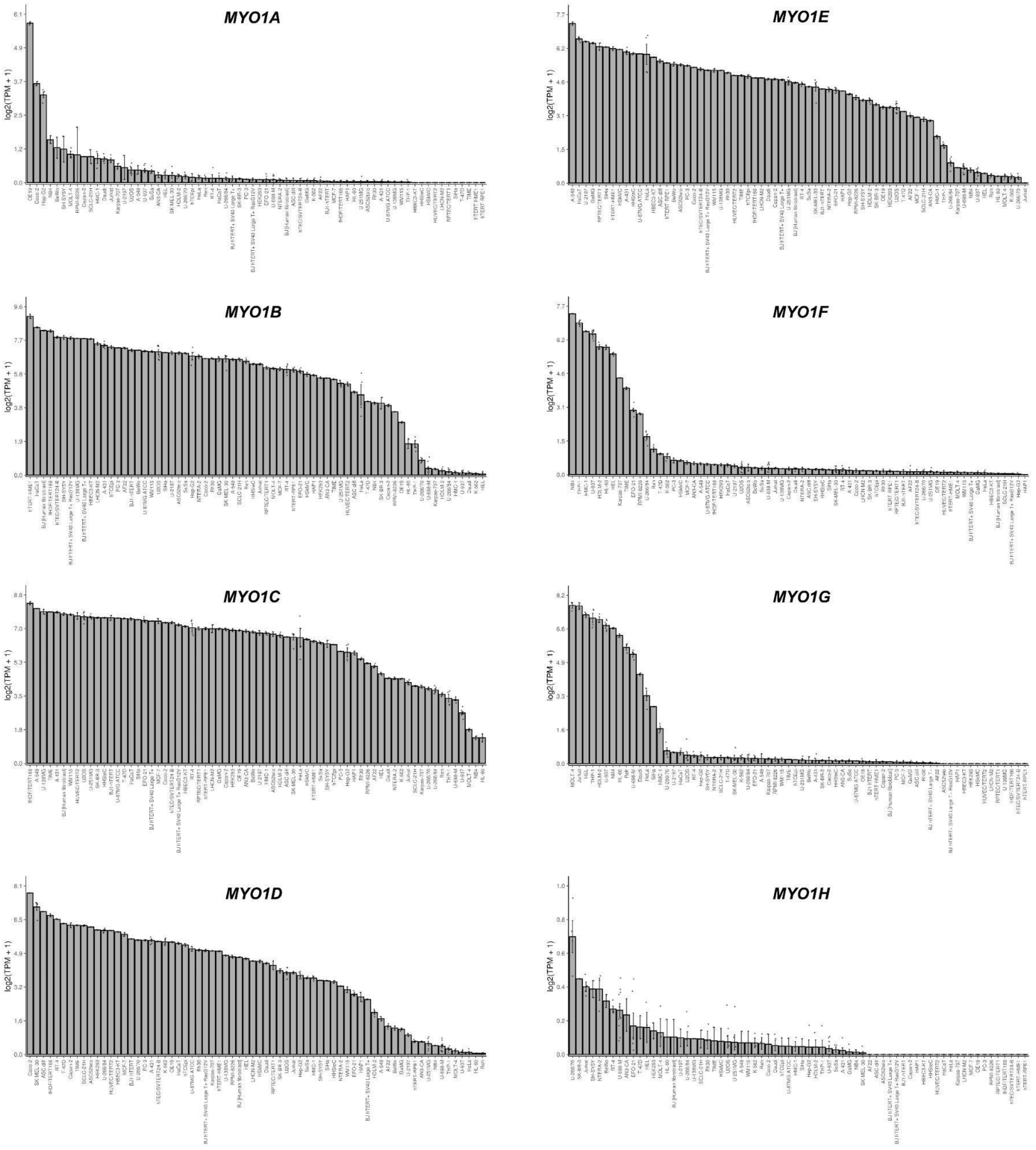

Supplementary Figure S3. The log2-transformed TPM values plotted for individual cell lines for each myosin I gene. Cell lines are ranged from the highest to the lowest log2(TPM+1) value.

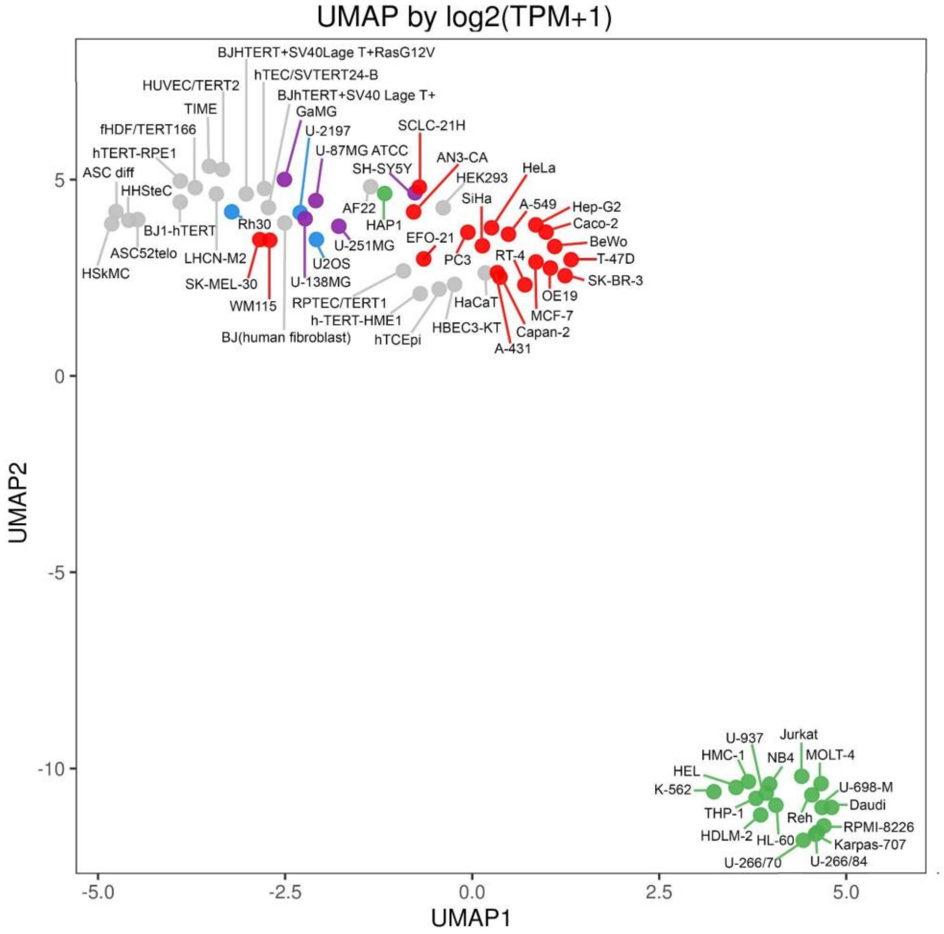

Supplementary Figure S4. UMAP analysis of the whole-transcriptome data of the 66 human cell lines. Each cell line is plotted as a dot, the choice of color for the dots is similar to Figure 1C and corresponds to the cell line annotation: non-malignant cell lines of different origin (grey), malignant carcinomas (red), malignant fibromas/sarcomas (blue), haematologic malignancies (green); malignant neuroblastomas/glioblastomas (purple).

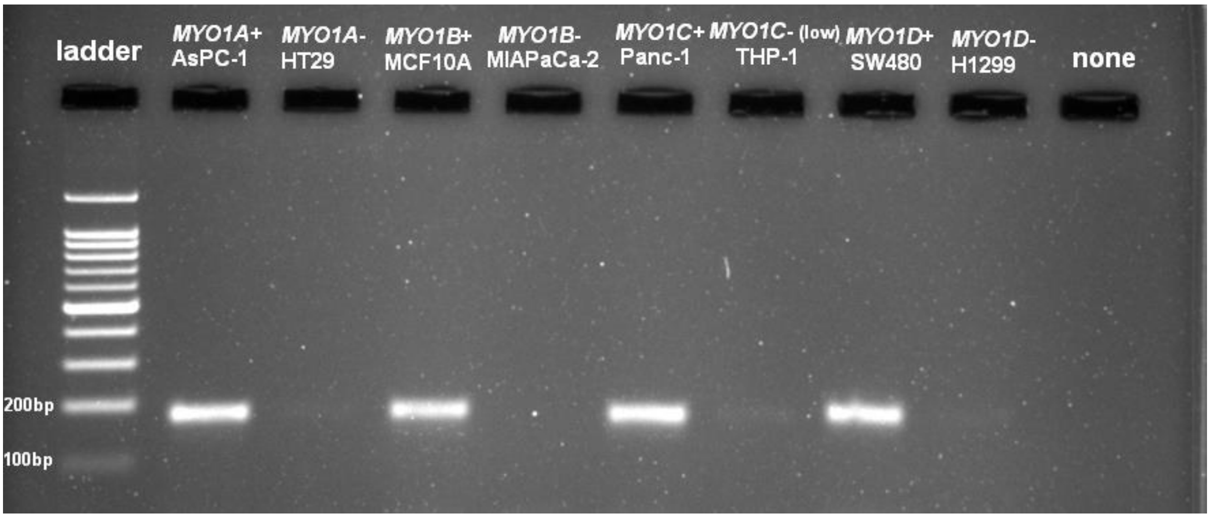

Supplementary Figure S5. The representative analysis of *MYO1A*-*MYO1D* PCR products by agarose electrophoresis. The wells from left to right contain the DNA specimens: DNA reference standards; *MYO1A*-positive cell line; *MYO1A*-negative cell line; *MYO1B*-positive cell line; *MYO1B*-negative cell line; *MYO1C*-positive cell line; *MYO1C*-negative (low) cell line; *MYO1D*-positive cell line; *MYO1D*-negative cell line; polymerase control (none).

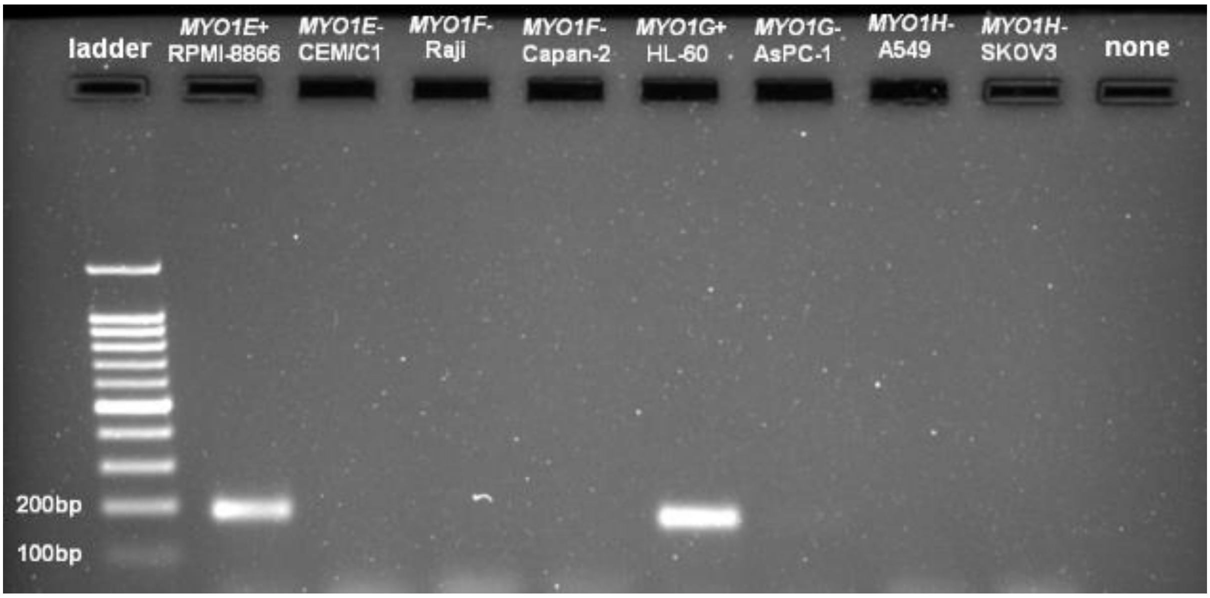

Supplementary Figure S6. The representative analysis of *MYO1E*-*MYO1H* PCR products by agarose electrophoresis. The wells from left to right contain the DNA specimens: DNA reference standards; *MYO1E*-positive cell line; *MYO1E*-negative cell line; *MYO1F*-negative cell line; *MYO1F*-negative cell line; *MYO1G*-positive cell line; *MYO1G*-negative cell line; *MYO1H*-negative cell line; *MYO1H*-negative cell line; polymerase control (none).

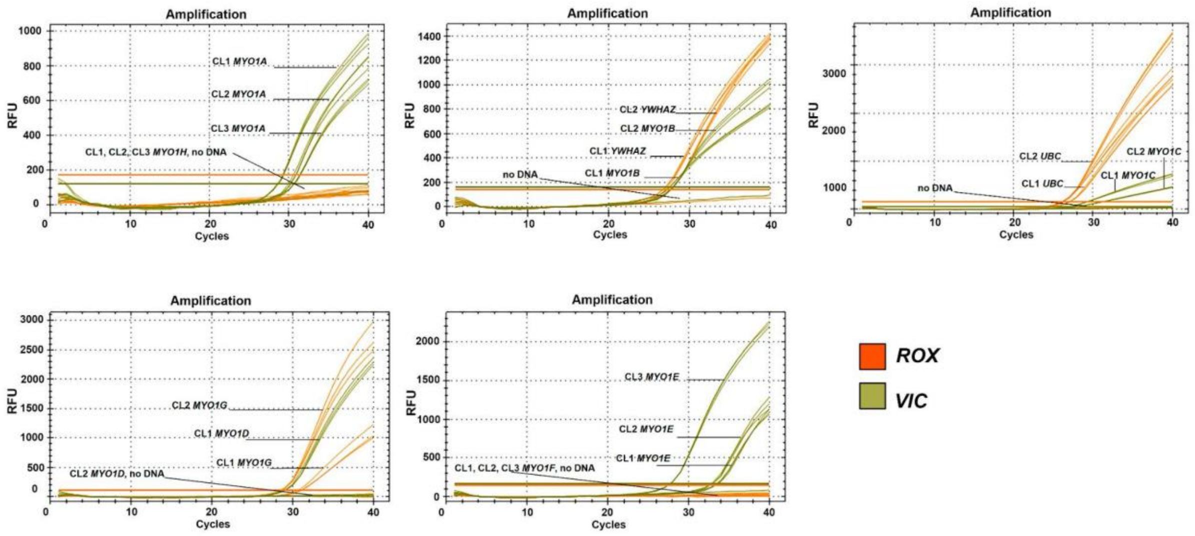

Supplementary Figure S7. The representative amplification curves of TaqMan multiplexed experiments. Shown are the *MYOA*+*MYOH* reaction for cell lines CL1, CL2; *YWHAZ*+*MYO1B* reaction for cell lines CL1, CL2; *UBC*+*MYO1C* reaction for cell lines CL1, CL2; *MYO1D*+*MYO1G* reaction for cell lines CL1, CL2; *MYOF*+*MYOE* reaction for cell lines CL1, CL2, CL3.

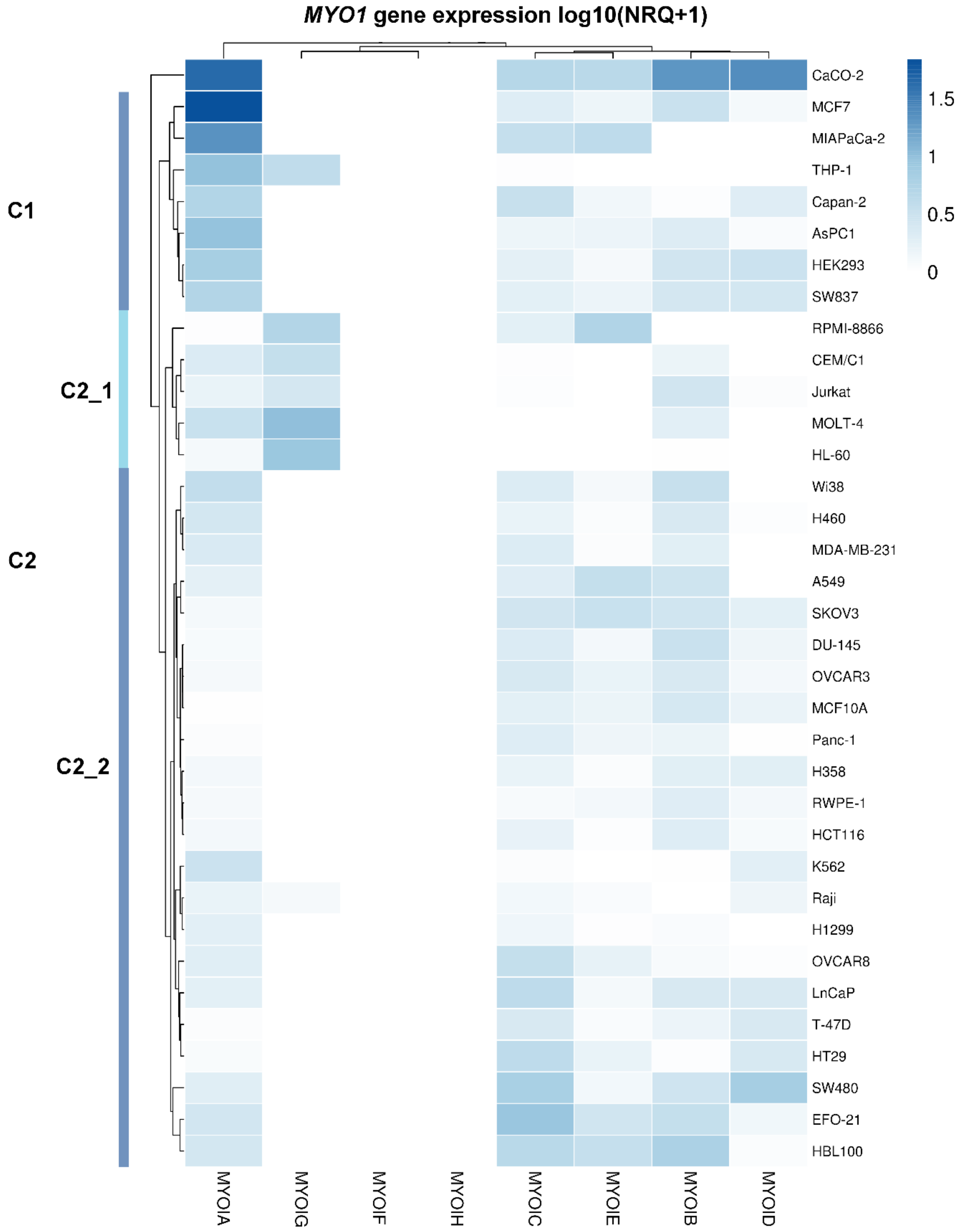

Supplementary Figure S8. The Ward clusterization analysis of *MYO1* expression in 35 cell lines assessed by qPCR. Each *MYO1* gene expression value is presented as a normalized relative quantity (NRQ) that was log10-transformed and plotted as a cluster map (the linear distance in the plot represents the Euclidean distance – a difference between specimens). Cluster C1 contains cell lines not enriched by type with elevated relative *MYO1A* expression. Cluster C2 is further subdivided into C2_1 and C2_2. Cluster C2_1 is enriched in haematopoietic cell lines while cluster C2_2 is not enriched by cell type.
