# Supplementary figures and images for: Myosin Class I Genes Follow a Cell Type-Specific Transcription Pattern in Human Haematopoietic Cell Lines
Source: Int J Mol Sci. 2026 Feb 12;27(4):1777. doi: 10.3390/ijms27041777 (PMC12940969; doi:10.3390/ijms27041777)

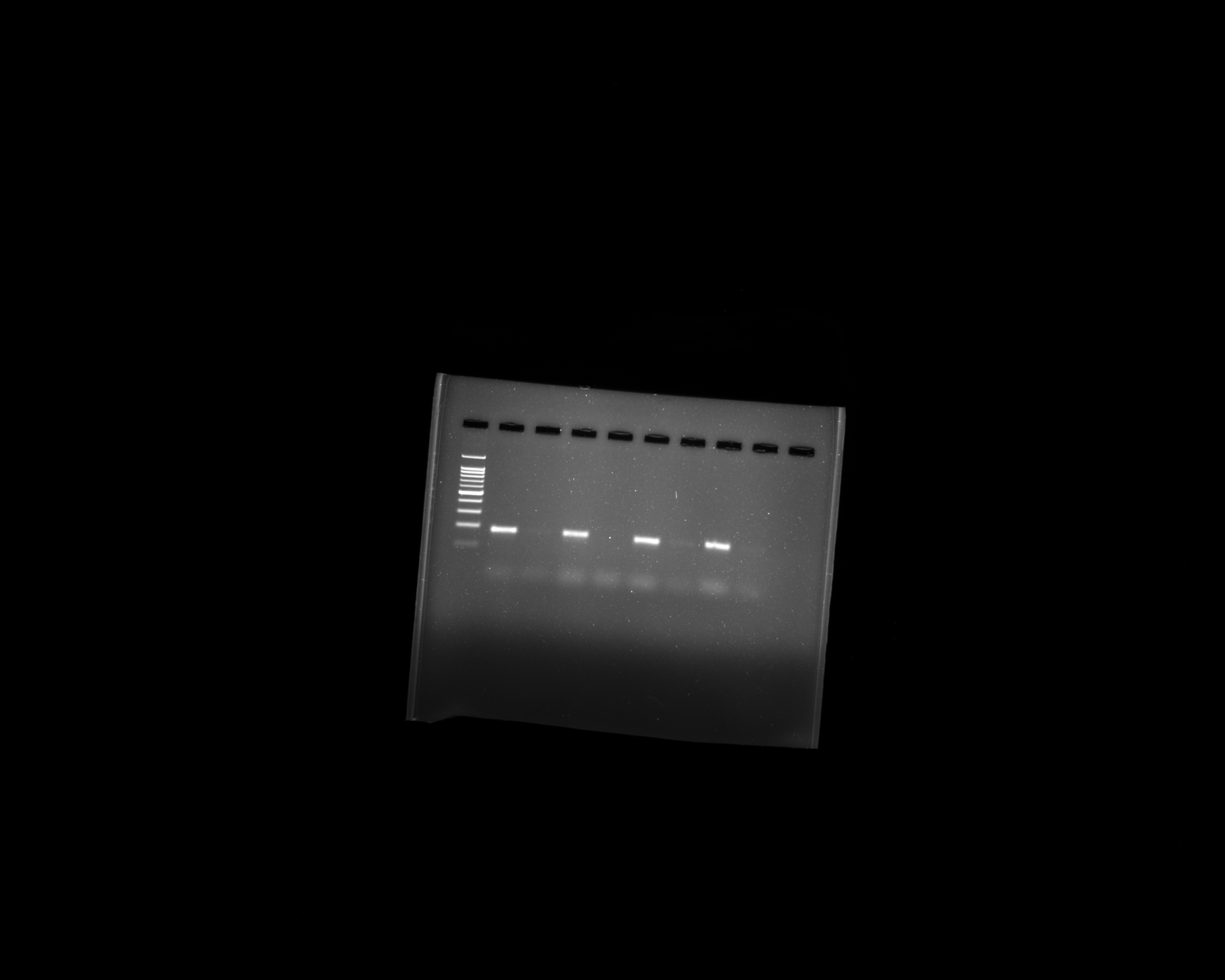

Supplement: Supplementary file 1 [file ijms-27-01777-s001.zip › Supplementary Data/Gel1_raw.tif]

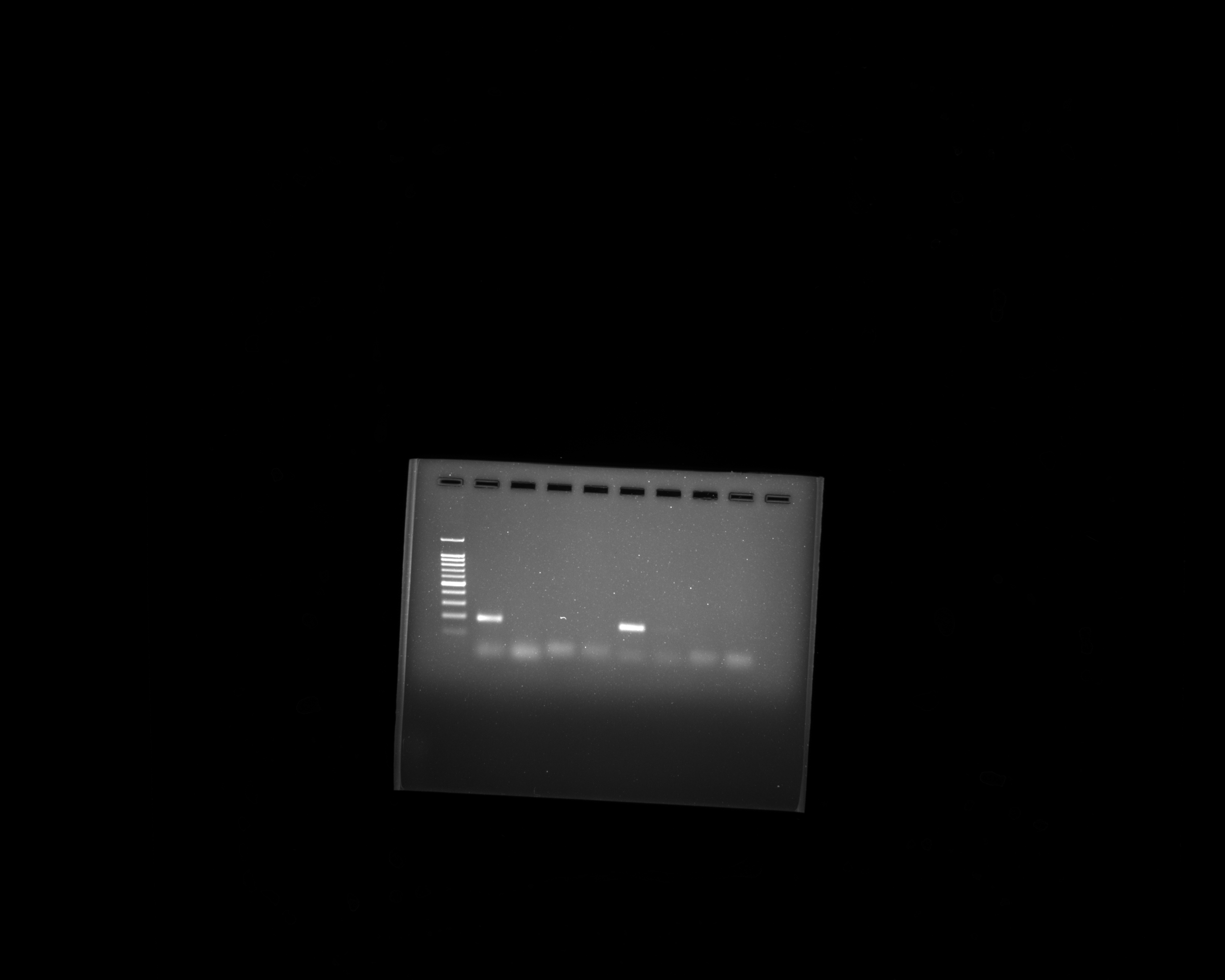

Supplement: Supplementary file 1 [file ijms-27-01777-s001.zip › Supplementary Data/Gel2_raw.tif]
